# Development of Tripolymeric Triaxial Electrospun Fibrous Matrices for Dual Drug Delivery Applications

**DOI:** 10.1038/s41598-020-57412-0

**Published:** 2020-01-17

**Authors:** Naveen Nagiah, Christopher J. Murdock, Maumita Bhattacharjee, Lakshmi Nair, Cato T. Laurencin

**Affiliations:** 1 Connecticut Convergence Institute for Translation in Regenerative Engineering, Farmington, Connecticut United States of America; 2 Raymond and Beverly Sackler Center for Biomedical, Biological, Physical and Engineering Sciences, Farmington, Connecticut United States of America; 3 Department of Orthopaedic Surgery, University of Connecticut Health Center, Farmington, Connecticut United States of America; 4 Department of Materials Science & Engineering, University of Connecticut, Storrs, Connecticut United States of America; 5 Department of Biomedical Engineering, University of Connecticut, Storrs, Connecticut United States of America; 6 Department of Chemical & Biomolecular Engineering, University of Connecticut, Storrs, Connecticut United States of America

**Keywords:** Biomedical engineering, Implants

## Abstract

Since the first work by Laurencin and colleagues on the development of polymeric electrospinning for biomedical purposes, the use of electrospinning technology has found broad applications in such areas of tissue regeneration and drug delivery. More recently, coaxial electrospinning has emerged as an important technique to develop scaffolds for regenerative engineering incorporated with drug(s). However, the addition of a softer core layer leads to a reduction in mechanical properties. Here, novel robust tripolymeric triaxially electrospun fibrous scaffolds were developed with a polycaprolactone (PCL) (core layer), a 50:50 poly (lactic-co-glycolic acid) (PLGA) (sheath layer) and a gelatin (intermediate layer) with a dual drug delivery capability was developed through modified electrospinning. A sharp increase in elastic modulus after the incorporation of PCL in the core of the triaxial fibers in comparison with uniaxial PLGA (50:50) and coaxial PLGA (50:50) (sheath)-gelatin (core) fibers was observed. Thermal analysis of the fibrous scaffolds revealed an interaction between the core-intermediate and sheath-intermediate layers of the triaxial fibers contributing to the higher tensile modulus. A simultaneous dual release of model small molecule Rhodamine B (RhB) and model protein Fluorescein isothiocynate (FITC) Bovine Serum Albumin (BSA) conjugate incorporated in the sheath and intermediate layers of triaxial fibers was achieved. The tripolymeric, triaxial electrospun systems were seen to be ideal for the support of mesenchymal stem cell growth, as shrinkage of fibers normally found with conventional electrospun systems was minimized. These tripolymeric triaxial electrospun fibers that are biomechanically competent, biocompatible, and capable of dual drug release are designed for regenerative engineering and drug delivery applications.

## Introduction

Conventional electrospinning technique produces uniaxial fibers with high surface area to volume ratios with extracellular matrix mimicking properties. Since the first work by Laurencin^1^ and colleagues on the development of polymeric electrospinning for biomedical purposes, the use of electrospinning technology has found broad applications in such areas of tissue regeneration, wound healing and drug delivery^2–25^. More recently, coaxial electrospinning has emerged as an important technique to develop scaffolds for regenerative engineering incorporated with drug(s)^26,27^. The coaxial electrospinning technique allows the feasibility of simultaneously encapsulating hydrophilic and hydrophobic drug moieties for efficient delivery at the targeted site (10). However, the addition of a softer core layer leads to a reduction in mechanical properties^28,29^. The lower elastic modulus of the coaxially electrospun fibers has also been attributed to the low viscoelasticity of encapsulated drug^29,30^.

Triaxially electrospun fibers for multiple drug release have been more recently described^31–35^. Different layers of triaxially electrospun fibers can be loaded with drugs in order to develop multi-drug delivery systems^31–35^. Studies have demonstrated that a controlled release of drug from a core layer can be achieved in the presence of an intermediate layer in the triaxial fibers^34,35^. Although release of active pharmaceuticals and model drugs have been investigated with an intermediate layer, attempts at creating simultaneous drug delivery from the sheath and intermediate layers of the triaxial electrospun fibers have been unexplored.

Our belief was that the addition of a strong core layer to triaxially electrospun fibers could be used to create biomechanically functional electrospun scaffolds while the sheath and intermediate layer could be used for consecutive or simultaneous release of multiple drugs. The goal of the present study was to design triaxially electrospun polycaprolactone (PCL) (core fiber layer), 50:50 poly (lactic-co-glycolic acid) (PLGA) (sheath layer) and gelatin (intermediate layer) fibers capable of release of multiple drugs simultaneously. The delivery of multiple biological factors has become a new and important goal in regenerative engineering and drug delivery applications (1). In our work multiple drug delivery was studied using a model small molecule rhodamine B (RhB) released from the sheath layer and a large molecule fluorescein isothiocynate (FITC) bovine serum albumin (BSA) conjugate in the intermediate layer. Differences in structural and mechanical properties after the addition of each layer were studied. Our overall hypothesis was that this novel tripolymeric triaxial electrospun fiber system would result in improved biomechanical properties. Our hypothesis also was that this novel electrospun fiber system could successfully deliver multiple model drugs.

## Materials

The polymer 50:50 Poly (DL-lactide-co-glycolide) ester terminated was obtained from DURECT Corporation (product name Lactel®, Birmigham, AL) and gelatin Type A was purchased from (MP Biomedicals LLC (Solon, OH). Rhodamine B was purchased from Acros Organics (New Jersey, USA). Polycaprolactone was purchased from Polysciences Inc. (Warrington, PA, USA). Chemical reagents were purchased from Sigma-Aldrich Inc. (St Louis, MO, USA) and Fisher Scientific, USA unless specified otherwise.

## Methods

### Electrospinning of polymer fibers

The apparatus used for obtaining triaxial fibers was developed in house. A core hydrophobic PCL polymer solution was passed through an inner needle of 22 G (0.71 mm in outer diameter and 0.41 mm in inner diameter), gelatin solution was passed through an intermediate needle of 15 G (1.372 mm in internal diameter and 1.829 mm in outer diameter) and PLGA(50:50) sheath solution was passed through a 10 G needle (2.692 mm in internal diameter and 3.404 mm in outer diameter). The needle gauges were maintained for uniaxial electrospinning (PLGA (50:50)) and coaxial (PLGA (50:50) (sheath) and gelatin (core) solutions. Polymer solutions were prepared by dissolving respective polymers in 1,1,1,3,3,3 hexafluoro-2-propanaol (HFP) at different concentrations; 25% w/v PLGA(50:50), 2% w/v gelatin and 1% w/v PCL. The solutions were loaded in 10 mL syringes with conducting needles. The needles were connected to the positive terminal of a high voltage ES30P 10 W power supply (Gamma High Voltage Research, Ormond Beach, FL). The core and/or intermediate polymer solutions were extruded at 0.5 mL/h and the sheath polymer solutions were extruded at 0.75 mL/h using syringe pumps (Pump 11 Plus, Harvard Apparatus, Boston, MA). The electric potential used was between 0.75–1.5 kV/cm. The fibers were deposited onto a grounded static aluminum substrate placed at a distance of approximately 10–25 cm perpendicular to the needle. To prepare drug loaded fibers, 0.25% w/v of rhodamine B (RhB) was added to 25% w/v PLGA(50:50) and 1% fluorescein isothiocynate (FITC) Bovine Serum Albumin (BSA) conjugate was added to 25% w/v PLGA(50:50) (in case of uniaxial electrospinning) or 2% w/v gelatin (in case of coaxial and triaxial electrospinning) before electrospinning. The obtained samples were stored at room temperature in desiccators until further use.

### Scanning electron microscopy

The aluminum substrates coated with the electrospun fibers were mounted on brass stubs and observed under a scanning electron microscope (JEOL JSM 6330 LV, USA) operating at an accelerating voltage of 5–20 kV. The diameters of 50 different fibers were measured in each of the cases using the UTHSCA Image tool to obtain their average diameter.

### Transmission electron microscopy

The internal structure of the uniaxial, coaxial and triaxial electrospun fibers were observed using an H7650 transmission electron microscope (JEOL JEM-2010 TEM,USA) operated at 80 kV. The samples for TEM were prepared by direct deposition of electrospun fibers on carbon-coated TEM grids.

### Thermal analysis

Thermogravimetric analysis (TGA) of the uniaxial, coaxial and triaxial electrospun fibers was performed using a universal TA Instruments TGA Q-500. Samples (5 mg) were heated at 10 °C min^−1^ in a temperature range of 0–600 °C using platinum crucibles. Differential Thermal Analysis was performed with differential thermal curves obtained from the TGA values.

Differential scanning calorimetry (DSC) analysis of the fibers was performed from 0 to 150 °C at 20 °C min^−1^ using a TA Instruments DSC Q 20. The instrument was calibrated using an indium standard, and the calorimeter cell was flushed with liquid nitrogen at 20 mL min^−1^.

### Tensile strength

Tensile strength of the electrospun membranes (50 mm × 6 mm × 0.25 mm) was tested using TA Q800 DMA instrument. Stress-strain measurements were carried out by equilibrating the chamber at 37 °C. The isothermal temperature was maintained for 10 seconds before a ramp force of 0.5 N/min was applied to a maximum of 18 N. Young’s modulus of the electrospun fibers was calculated from the slope values obtained from the stress strain graphs.

### Surface area

The surface areas of the electrospun fibrous scaffolds were measured with a Quantachrome NOVA 2000e unit by nitrogen adsorption at 77 K followed by outgassing at 30 °C using the Brunauer-Emmett-Teller (BET) method.

### *In vitro* biocompatibility

#### Cells isolation

The isolation procedure and study with the isolated cells was performed in accordance and approval with University of Connecticut Health Center Institutional Animal Care and Use Committee (IACUC) approved protocol. Rat adipose derived stem cells (ADSCs) were isolated as described earlier^36^. ADSCs were isolated from the inguinal fat pads of 7-week-old male Sprague Dawley rats. The rats were euthanized through CO_2_ inhalation followed by neck dislocation, according to University of Connecticut Health Center Institutional Animal Care and Use Committee (IACUC) approved protocol. The inguinal regions of the rats were shaved and sterilized by dipping the dead body in 70% ethanol. Then, the inguinal fat pads were isolated, using aseptic techniques, and weighed. The fat pads were collected in sterile Hank’s Balanced Salt Solution (HBSS) with Ca and Mg and 1% of anti-anti solution (10,000 units/mL of penicillin, 10,000 µg/mL of streptomycin, and 25 µg/mL of Gibco Amphotericin B) and washed at least 3 times. The fat piece was finely minced, collected in a falcon tube containing an equal volume of type II collagenase (0.1%) and HBSS, and incubated at 37 °C for 90 min under continuous shaking. Then, the type II collagenase was neutralized by adding equal volumes of DMEM-F12 containing 10% of FBS and 1% of anti-anti. The mixture was centrifuged at 1500 rpm for 10 min. The supernatant was removed and 2 ml of red blood cell lysis buffer (Sigma-Aldrich) was added to the cell pellet to lyse the red blood cells mixed in with the ADSCs. The cell pellet was then incubated for 2 min, centrifuged again under the same conditions, and finally re-suspended in DMEM-F12 (Gibco) containing 10% of FBS and 1% of anti-anti. Cells were counted and plated in 75 cm^2^ cell culture flasks maintained at 37 °C in a humidified incubator with 5% CO_2_. Culture media was changed after 48 hours, removing the non-adherent cells from the ADSCs attached to the flask. The culture media was changed every two days in order to maintain sub-confluent levels.

#### MTS assay

The electrospun scaffolds were exposed to UV light for 2 h to minimize contamination^37^. The ADSCs were seeded on the scaffolds (approximately 20,000 cells/cm^2^) and incubated at 37 °C and 5% CO_2_ for 72 hours. The culture medium was removed and a tetrazolium salt (3-(4,5-dimethylthiazol-2-yl)-5-(3-carboxymethoxyphenyl)-2-(4-sulfophenyl)-2H-tetrazolium], MTS; G3580, Cell Titer 96Aqueous One Solution Cell Proliferation Assay, Promega, USA) assay was performed as per the manufacturer’s protocol. Briefly, 20 μL of MTS solution was added to 100 μL of culture media. After incubation for 30 min at 37 °C, the absorbance of each well was measured at 490 nm.

#### Scanning electron microscopy with cells

To study cell adhesion and proliferation, the cell-seeded scaffolds were rinsed with PBS after 6 days of cell seeding and fixed using 10% neutral formalin buffer for 5 h at 4 °C. Dehydration of scaffolds was carried out through a series of graded alcohol solution and then dried. The fixed samples were sputter coated with gold for 30 seconds and mounted on brass stubs for scanning electron microscopy.

### Drug release studies

Three samples (1.5 mm × 1.5 mm) squares from each group were inserted into Conical Centrifuge Tubes (Thermo-Scientific) containing 25 mL of PBS pH 7.4. Then, 2 mL of buffer was aliquoted into MCT graduated tubes (Fisher Scientific) every two hours over a twenty-one day time period. The aliquoted samples were analyzed utilizing a Plate Reader (BioTek Synergy HT). The average drug loaded in the electrospun fibers were estimated by dissolving triplicate squares of electrospun fibers of the same dimensions in 1,1,1,3,3,3 hexafluoro-isopropanol and read at 630 nm for RhB and 540 nm for BSA-FITC respectively. Standard solutions for determining the cumulative drug loaded in electrospun fibers were prepared by dissolving known concentrations of the drug in 1,1,1,3,3,3 hexafluoro-isopropanol and read at 630 nm for RhB and 540 nm for BSA-FITC. The corresponding electrospun films of same dimension and approximate weight were dissolved in HFIP and used as control to determine the total loaded drug concentration. Similarly, the drug released into the PBS buffer was estimated by preparing standard drug solutions of known concentrations in PBS pH 7.4.

### Statistical analysis

All experiments were performed in triplicate and values were expressed as means ± standard deviations. All data were compared using Student’s t-test with *p* < 0.05 considered to be statistically significant.

## Results

### Electron microscopy of electrospun fibers

**Figure 1:**
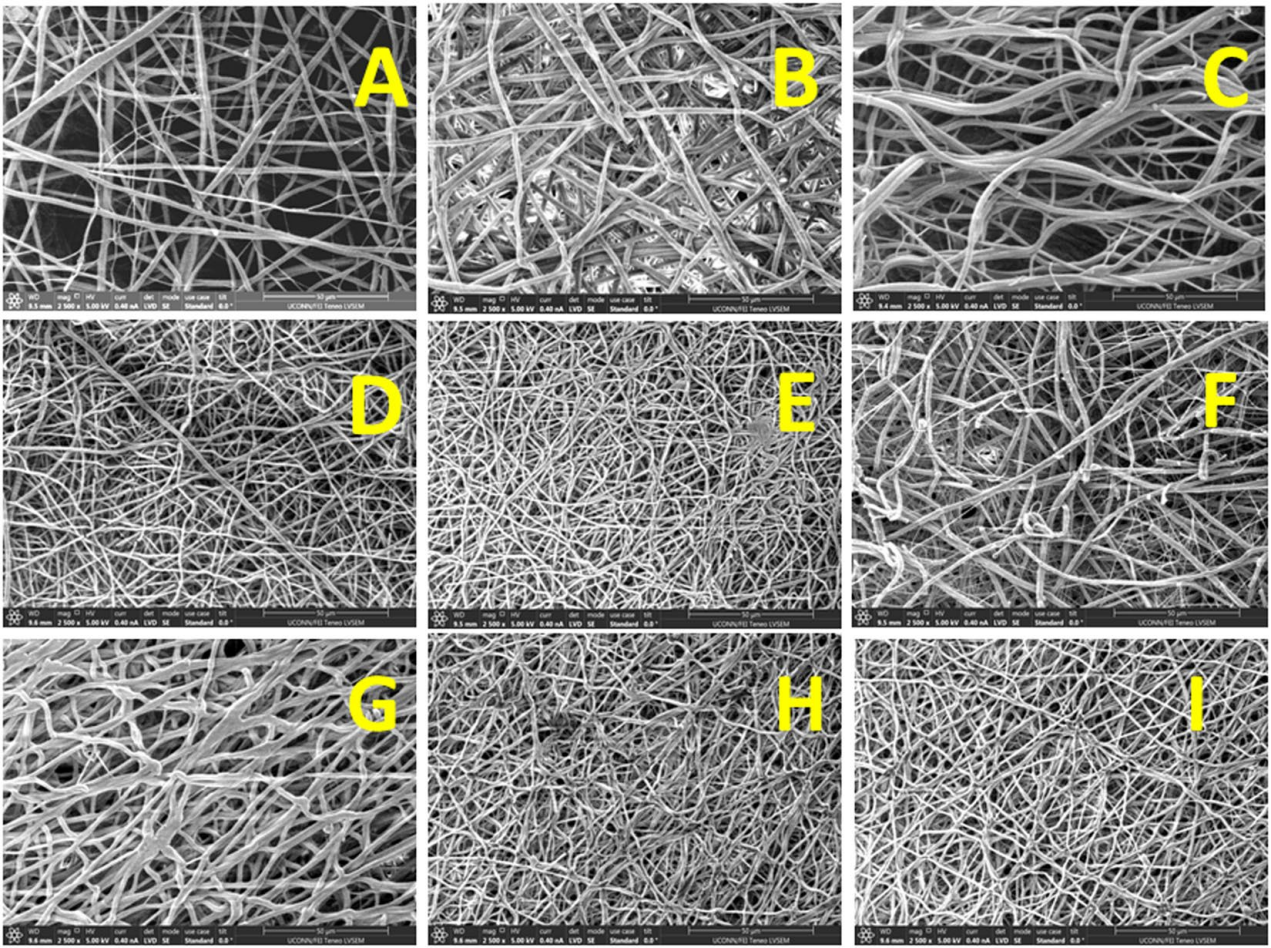
Scanning Electron microscopy of electrospun fibers (scale bar 50 µm). (**A**) Uniaxial PLGA(50:50) electrospun fibers. (**B**) Coaxial PLGA(50:50) (sheath) and gelatin(core) fibers. (**C**) Triaxial PLGA(50:50) (sheath), gelatin(intermediate) and PCL(core) fibers. (**D**) RhB loaded Uniaxial PLGA(50:50) electrospun fibers. (**E**) BSA-FITC loaded Uniaxial PLGA(50:50) electrospun fibers. (**F**) Coaxial PLGA(50:50) (sheath) and gelatin (core)- BSA-FITC electrospun fibers. (**G**) Coaxial PLGA(50:50)- RhB (sheath) and gelatin(core)- BSA-FITC electrospun fibers. (**H**) Triaxial PLGA(50:50) (sheath), gelatin(intermediate)- BSA-FITC and PCL(core) electrospun fibers. (**I**) Triaxial PLGA(50:50) (sheath) - RhB, BSA-FITC- gelatin(intermediate) and PCL(core) electrospun fibers.

Surface morphology of the electrospun fibers was determined using scanning electron microscopy (Fig. 1A–I). Figure 1A–C show the SEM images of uniaxial PLGA fibers, coaxial (PLGA sheath- gelatin core) fibers and triaxial (PLGA sheath-gelatin intermediate and PCL core) fibers respectively. Under an optimized flow rate of 0.75 mL/h for the sheath polymer PLGA and 0.5 mL/h for the core/intermediate gelatin and PCL polymers produced bead free continuous fibers. For uniaxial and coaxial electrospinning, the distance between the needle tip and collector was kept at 13 cm and the applied electric field ranged from 0.8–1.2 kV/cm. The distance was increased to 22 cm for triaxial electrospinning to avoid spraying and deformation of electrospun fibers (Supplementary Fig. S1). Table 1 shows the average fiber diameters of uniaxial, coaxial and triaxial fibers. The introduction of core (gelatin) in coaxial and core-intermediate (PCL-gelatin) layers in triaxial fibers led to a significant increase in average fiber diameters.

**Table 1:** Average diameter of various electrospun systems.

Electrospun system	Average fiber diameter(µm)
Uniaxial PLGA	1.46 ± 0.68
Uniaxial PLGA-RhB^*^	0.92 ± 0.3
Uniaxial PLGA-BSA-FITC^*^	1 ± 0.3
Coaxial PLGA-gelatin	1.82 ± 0.29
Coaxial PLGA-gelatin –BSA-FITC^†^	0.86 ± 0.63
Coaxial PLGA-RhB-gelatin- BSA-FITC^†^	2 ± 0.43
Triaxial PLGA-gelatin-PCL^‡^	1.88 ± 0.9
Triaxial PLGA-gelatin-BSA-FITC-PCL^‡^	1.26 ± 0.51
Triaxial PLGA-RhB-gelatin-BSA-FITC-PCL^‡^	1.16 ± 0.23

^*^Denotes p < 0.05 with Uniaxial PLGA as control.

^†^Denotes p < 0.05 with Coaxial PLGA-gelatin as control.

^‡^Denotes p < 0.05 with Triaxial PLGA-gelatin-PCL as control.

RhB and/or BSA-FITC incorporated uniaxial, coaxial and triaxial fibers are shown in Fig. 1D–I. The incorporation of RhB or BSA-FITC in uniaxial fibers led to a significant decrease in average fiber diameters. In coaxial fibers, the addition of BSA-FITC in the gelatin core led to a significant decrease in average fiber diameter. However, the incorporation of RhB in PLGA (50-:50) sheath along with BSA-FITC in gelatin core led to a significant increase in average fiber diameter. Incorporation of BSA-FITC and/or RhB in gelatin intermediate and PLGA (50:50) sheath led to a significant decrease in average fiber diameter in triaxial fibers.

**Figure 2:**
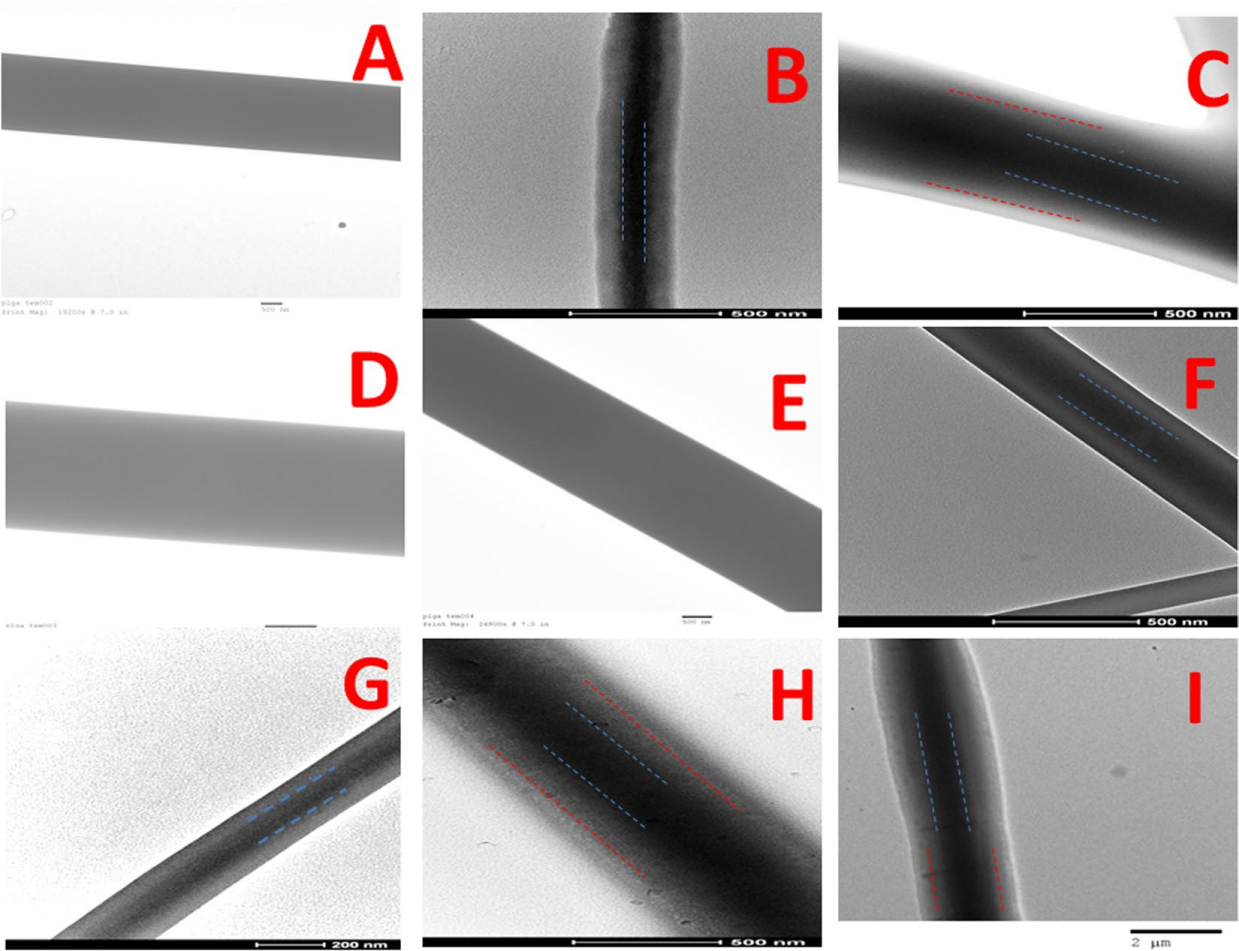
Transmission electron microscopy of electrospun fibers. (**A**) Uniaxial PLGA(50:50) electrospun fibers(scale bar 500 nm). (**C**) Coaxial PLGA(50:50) (sheath) and gelatin(core) fibers(scale bar 500 nm). (**C**) Triaxial PLGA(50:50) (sheath), gelatin(intermediate) and PCL(core) fibers(scale bar 500 nm). (**D**) RhB loaded Uniaxial PLGA(50:50) electrospun fibers(scale bar 500 nm). (**E**) BSA-FITC loaded Uniaxial PLGA(50:50) electrospun fibers(scale bar 500 nm). (**F**) Coaxial PLGA(50:50) (sheath) and gelatin(core)- BSA-FITC electrospun fibers(scale bar 500 nm). (**G**) Coaxial PLGA(50:50)- RhB (sheath) and gelatin(core)- BSA-FITC electrospun fibers(scale bar 200 nm). (**H**) Triaxial PLGA(50:50) (sheath), gelatin(intermediate)- BSA-FITC and PCL(core) electrospun fibers(scale bar 500 nm). (**I**) Triaxial PLGA(50:50) (sheath) - RhB,BSA-FITC- gelatin(intermediate) and PCL(core) electrospun fibers(scale bar 2 µm).

Figure 2 shows Transmission Electron Microscopy (TEM) images of the uniaxial, coaxial and triaxial fibers with/without BSA-FITC and/or RhB. The average diameter of PLGA (50:50) layer constituted approximately 55% of the thickness of coaxial fibers and reduced to approximately 27% of the thickness of triaxial fibers. The average diameter of the gelatin layer constituted approximately 45% of the coaxial fibers and 37% of triaxial fibers. The addition of RhB in the sheath layer and BSA-FITC in the intermediate/core layer of the coaxial and triaxial fibers was not found to significantly affect gelatin and PLGA (50:50) diameter percentages. Gelatin which formed the intermediate layer in the triaxial and core layer in coaxial electrospun fibers were not crosslinked as they were shielded through the PLGA(50:50) sheath.

### Thermal properties of electrospun fibers

**Figure 3:**
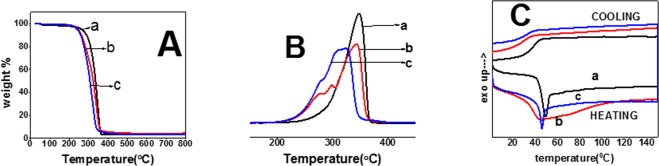
Thermal analysis of electrospun fibers. (**A**) Thermogravimetric analysis of: (a) Uniaxial PLGA(50:50) electrospun fibers. (b) Coaxial PLGA(50:50) (sheath) and gelatin(core) fibers. (c) Triaxial PLGA(50:50) (sheath), gelatin(intermediate) and PCL(core) fibers. (**B**) Differential thermal analysis of: (a) Uniaxial PLGA(50:50) electrospun fibers. (b) Coaxial PLGA(50:50) (sheath) and gelatin(core) fibers. (c) Triaxial PLGA(50:50) (sheath), gelatin(intermediate) and PCL(core) fibers. (**C**) Differential Scanning Calorimetry of: (a) Uniaxial PLGA(50:50) electrospun fibers. (c) Coaxial PLGA(50:50) (sheath) and gelatin(core) fibers. (c) Triaxial PLGA(50:50) (sheath), gelatin(intermediate) and PCL(core) fibers.

The thermal properties of uniaxial, coaxial and triaxial fibers were evaluated using TGA, DTA and DSC (Fig. 3 and Table 2). A single step weight loss between 200–400 °C was observed for uniaxial, coaxial and triaxial fibers (Fig. 3A) through TGA. The onset degradation temperature obtained from T_-5%_ values (critical significant weight loss of 5%) for uniaxial, coaxial and triaxial fibers was above 200 °C. The addition of the gelatin core in coaxial fibers and PCL-gelatin core-intermediate in triaxial fibers was found to decrease the T_-5%_ values. To confirm an interaction between the polymers, DTA values were obtained from TGA (Fig. 3B). A single distinct T_max_ peak for uniaxial, three peaks for coaxial and two peaks for triaxial fibers were observed. Since the peak ranges overlapped with the individual polymers (PCL, gelatin and PLGA (50:50)), DSC was performed to assess the interaction and miscibility of the polymers (Fig. 3C). The glass transition temperature of uniaxial fibers was observed at 48.32 °C. A single broad endothermic peak was observed for coaxial fibers ranging between 37–110 °C. In the case of the triaxial fibers, an endothermic peak at 46.1 °C was observed. The shift in glass transition temperature after the incorporation of gelatin core in coaxial and PCL-gelatin core-intermediate layers in triaxial fibers was observed. A shift in glass transition temperature of coaxial and triaxial fibers with respect to uniaxial fibers during the cooling cycle was also observed.

**Table 2:** Thermal, mechanical and surface properties of various electrospun systems.

	Uniaxial PLGA electrospun fibers	Coaxial (sheath PLGA and gelatin core) electrospun fiber	Triaxial (sheath PLGA, intermediate gelatin and core PCL) electrospun fibers
T_max_ (°C)	350.02	343.73, 279.28, 297.96	316.95, 281.21
T_-5%_ (°C)	265.57	250.17	244.12
Average Elastic modulus (MPa)	21.16 ± 6.77	13.78 ± 2.38	89.66 ± 27.8
Average Surface Area (m^2^/g)	3.41	1.17	0.34

### Tensile and surface properties of electrospun fibers

**Figure 4:**
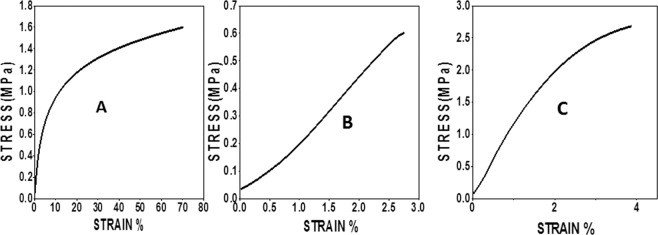
Dynamic Mechanical Analysis of electrospun systems at 37 °C. (**A**) Uniaxial PLGA(50:50) electrospun fibers. (**B**) Coaxial PLGA(50:50) (sheath) and gelatin(core) fibers. (**C**) Triaxial PLGA(50:50) (sheath), gelatin(intermediate) and PCL(core) fibers.

The tensile properties of the uniaxial, coaxial and triaxial fibers were studied using dynamic mechanical analysis at an isothermal temperature of 37 °C. Stress–strain curves and elastic moduli of the uniaxial, coaxial and triaxial fibers highlighted features that were intrinsic to the mechanical behaviors of these fibers (Fig. 4 and Table 2). For uniaxial fibers, the stress–strain curve can be divided into an elastic and strain-hardening region. The initial linear elastic region occurs up to 2% strain followed by the strain hardening. The initial linear elastic region is replaced by the toe region in coaxial and triaxial fibers. Coaxial and triaxial fibers exhibit a rather sudden failure while there was no break of the uniaxial fibers due to its elongation. Triaxial fibers exhibited maximum elastic modulus while coaxial exhibited the least. Addition of a gelatin core in coaxial fibers led to a significant decrease in elastic modulus, while introduction of a PCL core in triaxial fibers led to a significant increase in Young’s modulus when compared to uniaxial fibers.

The surface areas of uniaxial, coaxial and triaxial fibers were obtained through the Brunauer-Emmett-Teller (BET) method. Uniaxial fibers (3.41 m^2^/g) showed the highest surface area while triaxial fibers (0.34 m^2^/g) exhibited the lowest. The addition of a gelatin core in coaxial and a PCL-gelatin core-intermediate in triaxial fibers resulted in a corresponding decrease in surface area.

### Cell viability on electrospun fibers

**Figure 5:**
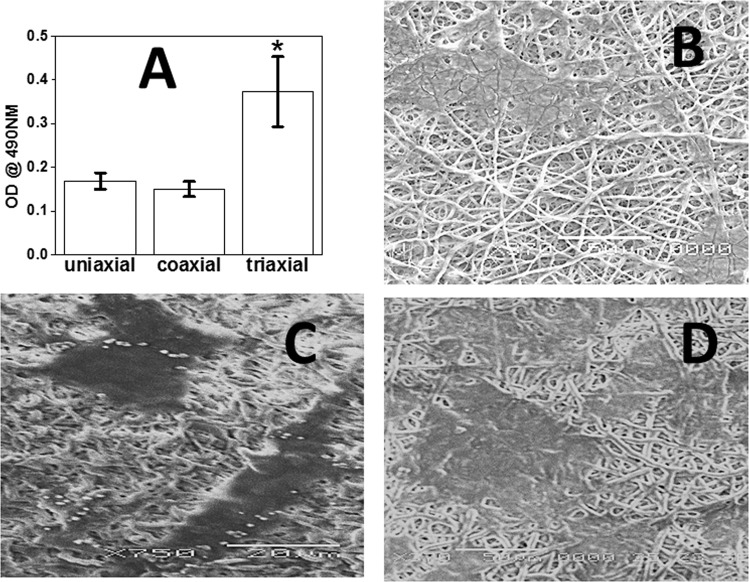
Biocompatibility of electrospun fibers. (**A**) MTS assay of electrospun systems after 3 days. (**B**) SEM of ADSCs on Uniaxial PLGA(50:50) electrospun fibers after 3 days. (**C**) SEM of ADSCs on Coaxial PLGA(50:50) (sheath) and gelatin(core) fibers after 3 days. (**D**) SEM of ADSCs on Triaxial PLGA(50:50) (sheath), gelatin(intermediate) and PCL(core) fibers after 3 days.

Bioactivity assessment of ADSCs in terms of attachment was achieved using SEM and viability on electrospun fibers using MTS assay. SEM was used to investigate the cell attachment on the electrospun fibers at day 3. Scanning electron microscopy images confirmed the adhesion of rat ADSCs on the uniaxial, coaxial and triaxial fibrous mats (Fig. 5B–D). SEM images showed no infiltration of the cells into the fibrous mats. To quantify the adherence and proliferation of ADSCs on uniaxial, coaxial and triaxial fibers MTS assay was performed. At 3 days following cell seeding, MTS values corresponding to cell numbers were significantly higher for triaxial fibers. Uniaxial and coaxial fibers showed no significant difference in cell numbers.

### Drug release from electrospun fibers

**Figure 6:**
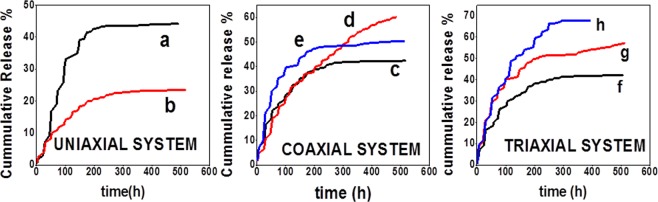
Drug release profile from electrospun fibers (Data shows mean ± standard deviation (n = 3)). (**a**) BSA-FITC release from uniaxial PLGA(50:50) electrospun fibers. (**b**) RhB release from uniaxial PLGA(50:50) electrospun fibers. (**c**) BSA-FITC release from coaxial PLGA(50:50) (sheath) and gelatin(core) electrospun fibers. (**d**) BSA-FITC release coaxial PLGA(50:50) (sheath) with RhB and gelatin(core) with BSA-FITC electrospun fibers. (**e**) RhB release from Coaxial PLGA(50:50) (sheath) with RhB and gelatin(core)with BSA-FITC electrospun fibers. (**f**) BSA-FITC release from triaxial PLGA(50:50) (sheath), gelatin(intermediate) with BSA-FITC and PCL(core) electrospun fibers. (**g**) RhB release from triaxial PLGA(50:50) (sheath) with RhB, gelatin(intermediate) with BSA-FITC and PCL(core) electrospun fibers. (**h**) BSA-FITC release from triaxial PLGA(50:50) (sheath) with RhB, gelatin(intermediate) with BSA-FITC and PCL(core) electrospun fibers.

The cumulative release of RhB and BSA-FITC from uniaxial, coaxial and triaxial fibers are shown in Fig. 6A–C. Release occurred in 2 stages and was an early higher release followed by steady controlled release. The cumulative release of RhB from uniaxial fibers (Fig. 6A.a) was significantly lower than BSA-FITC (Fig. 6A.b). BSA-FITC cumulative release was two times higher than RhB release from uniaxial fibers. In coaxial fibers (Fig. 6B), the cumulative release of RhB (Fig. 6B.e) was significantly higher when compared to its release from uniaxial fibers. BSA-FITC release from the gelatin core layer in coaxial fibers (Fig. 6B.d) was significantly higher when RhB was present in the sheath. Similarly, BSA-FITC cumulative release from the gelatin intermediate layer of triaxial fibers (Fig. 6C.h) was significantly higher when RhB was present in the sheath. RhB cumulative release from triaxial fibers (Fig. 6C.g) was significantly higher when compared to its release from uniaxial fibers. RhB cumulative release from the sheath layer of coaxial and triaxial fibers was approximately 50%, while BSA-FITC cumulative release from uniaxial (Fig. 6A.a), coaxial (Fig. 6B.c) and triaxial (Fig. 6C.f) without RhB was approximately 40%. Hence, the addition of RhB in the sheath layer of coaxial and triaxial fibers affected the cumulative release of BSA-FITC.

## Discussion

Triaxial electrospinning of scaffolds has been achieved in the past with only two polymers or with the same polymer at different concentrations in different layers^31–35^. A novel tripolymeric system of triaxial fibers, as presented here, would aid in achieving a modulated simultaneous release from the sheath and intermediate layers of the triaxial fibers. Electrospinning of polymer solutions and emulsions requires the optimization of various parameters (concentration, viscoelasticity, molecular weight, degree of entanglement, electrical conductivity, and surface tension)^38–41^. The synthetic polymers PLGA and PCL have been electrospun using solvents such as dichloromethane and chloroform^38^. Due to higher conductivity 1, 1, 1,3,3,3 hexafluorisopropanol (HFP) has been widely used to electrospin natural polymers (11). In addition, HFP also reduces surface tension of the polymer solution thereby reducing the concentration of polymer solution used for bead free electrospinning^26^. Hence HFP was chosen as the optimal solvent for electrospinning PLGA (50:50), gelatin and PCL fibers. The increase in diameter of coaxial fiber over uniaxial fibers has been attributed to the presence of gelatin core solution that caused a difference in charge relaxation time and viscosity of the coaxial spinning solution^27^. During triaxial electrospinning, when PCL polymer solution was introduced, a further decrease in charge relaxation time occurred due to the increase in polymer solution volume. This led to a further increase in fiber diameter. Similarly, interactive sheath-intermediate and core-intermediate layers were formed in triaxial fibers. The increase in fiber diameter corresponded to a decrease in surface area of the electrospun fibers^26^. The increase in solvent volume for electrospinning also led to incomplete evaporation of solvent during triaxial electrospinning which resulted in spraying during electrospinning (Supplementary Fig. 1). Increasing the distance between the tip and collector to 22 cm for triaxial electrospinning allowed the formation of spray free continuous fibers. Except for the coaxial electrospun system with both drugs the addition of RhB has led to a decrease in average fiber diameter. The difference in the charge densities and viscosities of the polymer solution led to an abrupt increase in average diameter^39^. In case of triaxial fibers, the addition of PCL in the core layer and increase in distance between the tip and collector played a vital role in average fiber diameter reduction.

Thermal properties of the electrospun fibers were used to assess the miscibility and interaction of the polymer(s) after electrospinning. The weight loss between 200 and 400 °C was due to the chain scission of ester linkages^39^. In addition to the T_max_ peaks of PLGA (50:50) and gelatin in coaxial fibers the third peak corresponds to the interactive core-sheath layer^26^. A similar effect of core-sheath interaction has been observed earlier by Naveen *et al*.^26^. The shift from a sharp endothermic peak (48.32 °C) in uniaxial fibers to a broad endothermic peak (37–110 °C) as observed from DSC of coaxial fibers confirms the interaction between the sheath and core polymers. The broad endothermic peak of gelatin observed between 50–150 °C (Supplementary Fig. 3) corresponds to denaturation and water loss. Similarly, in triaxial fibers, two broad T_max_ peaks corresponds to the interactive core-intermediate and sheath-intermediate layers. The shift in single endothermic peak to 46.1 °C as observed from DSC of triaxial fibers further confirms the presence of interactive layers of miscible semi-crystalline PCL (core)-gelatin (intermediate) and sheath PLGA(50:50)-gelatin(intermediate). The evaporation of solvent from the core and/or intermediate layer in coaxial and triaxial electrospinning caused expansion of core and/or intermediate layers of coaxial and triaxial fibers which resulted in the formation of the interactive layers and thinning of the PLGA (50:50) sheath layer^42–45^.

The elastic modulus of the electrospun fibers were significantly affected due to the coaxial and triaxial electrospinning process. Inclusion of a gelatin core in the coaxial fibers led to a significant thinning of PLGA (50:50) which led to a corresponding decrease in elastic modulus of the coaxial fibers^46^. Moreover, the elastic modulus of core non-interactive gelatin was significantly lower than the sheath PLGA (50:50) which further led to a decrease in elastic modulus. In spite of the thinning of the sheath PLGA (50:50), triaxial fibers showed significant increase in elastic modulus over coaxial fibers. This has been attributed to the absence of the non-interactive gelatin core and the interactive core-intermediate and sheath-interactive layers in triaxial fibers. Additionally, the thinning of intermediate layer in triaxial fibers also contributed to the increase in elastic modulus seen. The enhanced mechanical properties of these triaxially electrospun scaffolds increases its potential as a suitable scaffolds for bone regeneration^6^.

One of the major drawbacks of uniaxial PLGA (50:50) electrospun fibrous scaffolds is its shrinking effect in cell culture media^47^. Uniaxially electrospun fibers shrunk to nearly 80% its original dimensions while coaxially electrospun fibers shrunk to nearly 60% its original dimensions. The addition of a PCL core in the triaxial structures significantly decreased the shrinking of the fibrous scaffold. The reduction in shrinking presumably allowed higher number of cells to adhere on the triaxial fibers which resulted in significantly higher MTS activity.

The pattern of release of RhB and BSA-FITC from different electrospun systems is shown in Supplementary Information Figs. 4–6. A slow release of RhB from uniaxial PLGA (50:50) fibers has been reported earlier^48,49^. A higher release of BSA-FITC from uniaxial fibers in comparison with RhB was due to its higher solubility in water^50^. BSA-FITC is more hydrophilic and larger molecule than RhB thereby leading to higher water retention in electrospun fibers. A higher release of BSA-FITC from core with comparison to RhB from sheath of coaxial fibers was due to the increase in hydrophilicity and water retention. Hydrophilicity and water retention play an important role in drug release kinetics^48,51^. In case of coaxial and triaxial electrospun fibers, the addition of RhB in the sheath increased the water retention capacity of the sheath concurrently leading to a higher release of BSA-FITC from core/intermediate layers. The presence of hydrophilic RhB in sheath of coaxial fibers led to an increase in hydrophilicity of the fibers which resulted in higher water retention^51,52^. A similar lower release of small molecules from the sheath layer when loaded with large molecules in the core layer has been reported earlier^53^. A higher release of RhB from sheath of coaxial and triaxial fibers when compared to its release from uniaxial fibers was due to the thinning of PLGA (50:50) sheath layer.

The use of small molecules for regenerative engineering applications has been largely overlooked due to its rate limiting factor of triggering different signaling cascades^54^. The use of small molecules along with large molecules in a coaxial fibrous system for exhibited a synergistic effect which led to enhanced regeneration^53^. In addition to providing a structural support for infiltrating cells, this newly designed tripolymeric triaxially electrospun system could enable providing adequate doses of small and large molecules. The model small and large molecules can be substituted with suitable drugs for desired delivery at the targeted site.

In summary, electrospinning technology was used to develop next generation tripolymeric triaxially electrospun fibers of PLGA (50:50) sheath layer, gelatin intermediate layer and PCL core layer. Incorporation of model small (RhB) and large (BSA-FITC) molecules was performed throught the loading of sheath and intermediate layers. Thermal studies confirmed the interaction of core-intermediate and sheath-intermediate layers. A superior elastic modulus with controlled release of a both model small and large molecule was achieved with the tripolymeric triaxial electrospun fibers in comparison with its uniaxial and coaxial counterparts. The strengthening was due to the presence of good affinity between different layers of the triaxial fibers. The improved mechanical properties of the triaxially electrospun scaffold displays promising progress in regenerative engineering applications. The presence of the small molecule, RhB in the sheath layer significantly improved the release of the large molecule BSA-FITC. These tripolymeric triaxially electrospun mechanically enhanced matrices will be important for use in applications of regenerative engineering and drug delivery system.

## Supplementary information

Supplementary information
