# Supplementary material for: Development of Tripolymeric Triaxial Electrospun Fibrous Matrices for Dual Drug Delivery Applications
Source: Sci Rep. 2020 Jan 17;10:609. doi: 10.1038/s41598-020-57412-0 (PMC6969175; doi:10.1038/s41598-020-57412-0)
Supplement: Supplementary file 1 — Supplementary information [file 41598_2020_57412_MOESM1_ESM.docx]

**Development of Tripolymeric Triaxial Electrospun Fibrous Matrices for Dual Drug Delivery Applications**

*Naveen Nagiah^1^, Christopher J. Murdock^1^, Maumita Bhattacharjee^1^, Lakshmi Nair^1, 2, 5, 4^, Cato T. Laurencin^1, 2, 3,4,5,6,*^*

^1^Connecticut Convergence Institute for Translation in Regenerative Engineering, Farmington, Connecticut, United States of America

^2^Raymond and Beverly Sackler Center for Biomedical, Biological, Physical and Engineering Sciences, Farmington, Connecticut, United States of America

^3^Department of Orthopaedic Surgery, University of Connecticut Health Center, Farmington, Connecticut, United States of America

^4^Department of Materials Science & Engineering, University of Connecticut, Storrs, Connecticut, United States of America

^5^Department of Biomedical Engineering, University of Connecticut, Storrs, Connecticut, United States of America

^6^Department of Chemical & Biomolecular Engineering, University of Connecticut, Storrs, Connecticut, United States of America

***Corresponding author:**

**Cato T. Laurencin, M.D., Ph.D.**

University Professor

Albert and Wilda Van Dusen Distinguished Professor of Orthopaedic Surgery

Professor of Chemical and Biomolecular Engineering

Professor of Materials Science and Engineering

Professor of Biomedical Engineering

 Director, The Raymond and Beverly Sackler Center for Biomedical, Biological, Physical and Engineering Sciences

 Chief Executive Officer,

The Connecticut Convergence Institute for Translational in Regenerative Engineering

The University of Connecticut

UConn Health
263 Farmington Avenue L7036
Farmington, CT 06030

Phone: 860-679-4086

Email: [laurencin@uchc.edu](mailto:laurencin@uchc.edu)

| **S.No.** | **Sample** | **Outer sheath** | **Intermediate layer** | **Inner core** |
| --- | --- | --- | --- | --- |
| 1. | Uniaxial | PLGA(50:50) | - | - |
| 2. | Uniaxial RhB | PLGA(50:50) with 0.25% w/v RhB | - | - |
| 3. | Uniaxial BSA-FITC | PLGA(50:50) with 1% w/v BSA-FITC | - | - |
| 4. | Coaxial | PLGA(50:50) | - | Gelatin |
| 5. | Coaxial BSA-FITC | PLGA(50:50) | - | Gelatin with 1% w/v BSA-FITC |
| 6. | Coaxial both | PLGA(50:50) with 0.25% w/v RhB | - | Gelatin with 1% w/v BSA-FITC |
| 7. | Triaxial | PLGA(50:50) | Gelatin | PCL |
| 8. | Triaxial BSA-FITC | PLGA(50:50) | Gelatin with 1% w/v BSA-FITC | PCL |
| 9. | Triaxial both | PLGA(50:50) with 0.25% w/v RhB | Gelatin with 1% w/v BSA-FITC | PCL |

**Table S1. List of electrospun fibers developed for study**


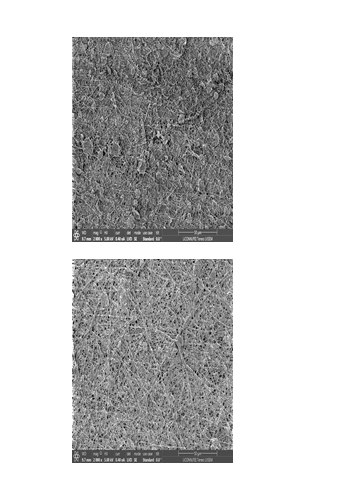


**Figure 1. SEM of triaxial fibers spun between 13-15cm**

## ATR-FTIR Spectroscopy

Attenuated total reflection-Fourier transform infrared spectroscopy (ATR-FTIR) measurements were carried out on peeled fibrous membranes using a Nicolet 6700 FTIR spectrometer (Thermo Fisher Scientific, USA) equipped with a diamond ATR crystal. Typically, 30 scans were signal-averaged to reduce spectral noise. The spectrum of the samples was recorded from 600 to 4000 cm^–1^.

#### The ATR-FTIR spectra of electrospun fibers are shown in [Figure 2](https://pubs.acs.org/doi/full/10.1021/acs.langmuir.5b03177#fig2)below. The characteristic absorption band between 1700 and 1750 cm^–1^ in all electrospun polymer systems is mainly due to the ester carbonyl group (C═O).^[1]^ Characteristic peaks at 1454 cm^-1^ correspond to methyl group C–H stretching while two peaks at 1426 and 1383 cm-1 correspond to wagging vibrations from saturated C–H bonds. Peaks at 1271, 1174, 1130, 1085 and 1051 cm1 represent C–O bonds.^[20]^ The peaks observed in the range of 1225–1280 cm^–1^ correspond to O—C—O symmetric and asymmetric stretching, while vibrations at 2925 and 2850 cm^–1^ correspond to C—H stretching bands of the CH_2_ and CH_3_ groups confirming the presence of the interactions among the electrospun fibers.^[4]^ The interaction between the fibrous layers is apparent only in the coaxial and triaxial fibers and completely absent in the uniaxial electrospun samples. The weak amine and amide peaks corresponding to rhodamine B and BSA-FITC were found to be between 1500-1550 cm^-1^ respectively. The presence of drugs hinders the interaction between the layers in coaxial and triaxial systems. Rhodamine B peaks are not apparent as their respective concentrations were overshadowed by the large polymers. Moreover, rhodamine B with unmodified PLGA have been undetectable through FTIR in the recent past [21]. Larger molecule bovine serum albumin peaks are not apparent as major volume of the molecules might be trapped within the fibrous scaffolds due to which no characteristic peaks were observed for the corresponding moieties with a high encapsulation efficiency. Since ATR-FTIR measures only the reflectance from a film surface, the degree of interaction between the intermediate-sheath/core–sheath and intermediate-core polymers could not be discerned.
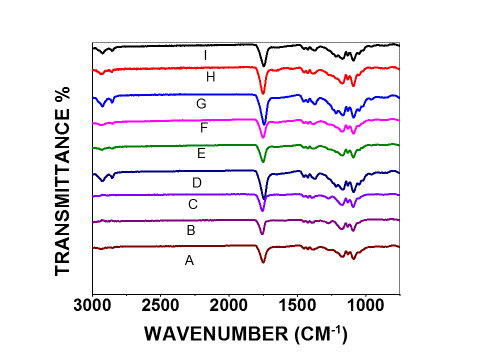


Figure 2. ATR-FTIR spectroscopy of electrospun fibers

1. Uniaxial PLGA(50:50) electrospun fibrous scaffold
2. Rhodamine B loaded Uniaxial PLGA(50:50) electrospun fibers
3. BSA-FITC loaded Uniaxial PLGA(50:50) electrospun fibers
4. Coaxial PLGA(50:50) (sheath) and gelatin(core) fibrous scaffold
5. BSA-FITC loaded Coaxial PLGA(50:50) (sheath) and gelatin(core) electrospun fibers
6. Rhodamine B and BSA-FITC loaded Coaxial PLGA(50:50) (sheath) and gelatin(core) electrospun fibers
7. Triaxial PLGA(50:50) (sheath) , gelatin(intermediate) and PCL(core) fibrous scaffold
8. BSA-FITC loaded Triaxial PLGA(50:50) (sheath) , gelatin(intermediate) and PCL(core) electrospun fibers
9. Rhodamine B and BSA-FITC loaded Triaxial PLGA(50:50) (sheath) , gelatin(intermediate) and PCL(core) electrospun fibers

**Figure 3. DSC of PCL and gelatin**


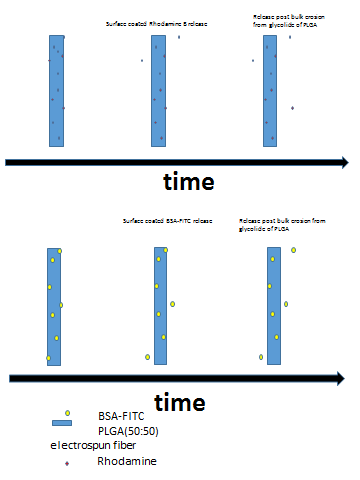


**Figure 4. Drug release mechanism from uniaxial system**


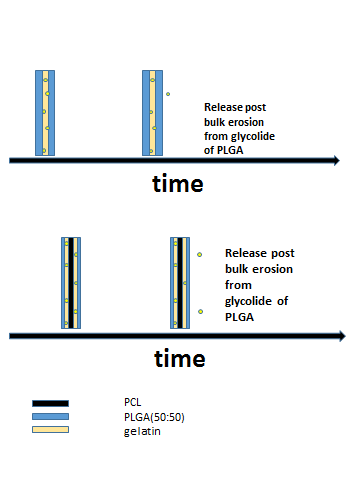


**Figure 5. Drug release from BSA-FITC conjugate alone loaded electrospun coaxial and triaxial system**


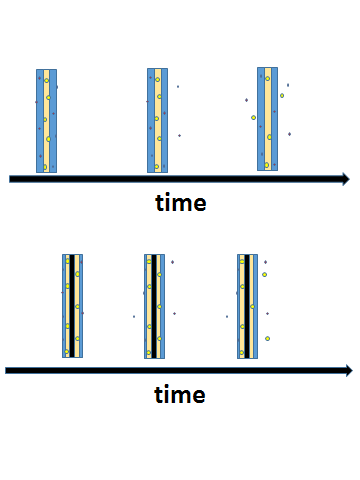


**Figure 6. Drug release mechanism from both BSA-FITC and RhB loaded coaxial and triaxial ystem**
